# Editorial: Community series-extremophiles: microbial genomics and taxogenomics, volume II

**DOI:** 10.3389/fmicb.2024.1371210

**Published:** 2024-01-31

**Authors:** André Antunes, Rafael R. de la Haba, Mohamed Jebbar, Brian P. Hedlund

**Affiliations:** ^1^State Key Laboratory of Lunar and Planetary Sciences, Macau University of Science and Technology, Taipa, Macau SAR, China; ^2^Macau Center for Space Exploration and Science, China National Space Administration (CNSA), Taipa, Macau SAR, China; ^3^China-Portugal Belt and Road Joint Laboratory on Space and Sea Technology Advanced Research, Virtual Lab; ^4^Department of Microbiology and Parasitology, Faculty of Pharmacy, University of Sevilla, Sevilla, Spain; ^5^Univ Brest, CNRS, Ifremer, EMR 6002 BIOMEX, Unité Biologie et Écologie des Écosystèmes Marins Profonds BEEP, Plouzané, France; ^6^School of Life Sciences, University of Nevada, Las Vegas, NV, United States; ^7^Nevada Institute of Personalized Medicine, University of Nevada, Las Vegas, NV, United States

**Keywords:** extremophiles, genomics, taxonomy, metagenomics, astrobiology, space microbiology, biodiversity

## Introduction

The importance of extreme environments and their microbial inhabitants has been gaining visibility. Such environments account for most of Earth's habitable zone by volume (Gold, 1992; Charette and Smith, 2010) and they are sources of valuable new strains and bio-products that will fuel the bioeconomy and will provide new solutions to the current challenges we face in the in areas of human health, energy, environment, and agriculture (Antunes et al., 2016; Corral et al., 2019). Developments in extremophile microbiology are increasingly linked with technological improvements in sequencing, bioinformatics, and other upcoming technologies that are opening new paths for scientific breakthroughs and promises to change our understanding of life on Earth. The evolution and physiology of extremophiles remain relevant to investigate the universal ancestor and the limits of life (Merino et al., 2019). The study of extremophiles is also vital to both Astrobiology and Space Microbiology and will support humanity's next steps into space (e.g., Antunes and Meyer-Dombard, 2023; Simões et al., 2023).

Within this setting, we hosted the special Research Topic entitled *Extremophiles: Microbial genomics and taxogenomics* (de la Haba et al., 2022). Based on its success, we then launched a second volume as the start of a community series dedicated to this topic. Our new volume includes 14 manuscripts, covering a wide range of areas within the field, with contributions on: (i) the discovery of new taxa and genomic-based analysis of their properties; (ii) new community-based studies in previously overlooked extreme and poly-extreme environments; and (iii) new insights into high taxonomic ranks of extremophiles. The volume was well received, with a total of 77 contributing authors from institutions in 14 different countries spread across the globe, namely Australia, Belgium, Botswana, China, Ethiopia, Germany, India, Italy, Poland, Russia, Spain, Sweden, United Kingdom, and United States of America. At the time of writing, over 190,000 views and downloads have been recorded for both volumes of the series.

## New taxa and their properties

The isolation of new extremophiles is vital within this field as highlighted by several contributions to this volume describing new taxa and the use of genomics to uncover some of their remarkable properties.

Two articles on hypersaline soils (Odiel Saltmarshes Natural Area, Spain) describe new taxa from this previously overlooked type of environment. In the first article, Galisteo et al. (a) describe the novel species *Fodinibius salsisoli*. Comparative genomics revealed that the species of the genera *Aliifodinibius* and *Fodinibius* belong to the same genus, so the authors propose the reclassification of the species of the former into the single genus *Fodinibius*. Further analysis highlighted abundant but still uncultured representatives of the family *Balneolacea* in this environment and their potential role as a source of biotin for other organisms. In the second publication from this group, Galisteo et al. (b) describe the new genus *Terrihalobacillus*, and the new species *Terrihalobacillus insolitus* and *Aquibacillus salsiterrae*, both based on several isolates. The genomic features of these new taxa were also analyzed, and both were suggested to belong to the rare biosphere of these environments.

An additional study by García-Roldan et al., also focusing on high-salinity environments in Spain (a solar saltern from Isla Cristina, Huelva), describes a new species of *Natronomonas*, named *Natronomonas aquatica*. Further genome-based phylogenetic and metabolic analysis of this genus confirmed its phylogenetic coherence, indicated a heterotrophic lifestyle and versatile nitrogen metabolisms, and suggested its ubiquity in mid- to high-salinity environments.

The contribution by Zavarzina et al. focuses on haloalkaliphiles inhabiting soda lakes in Mongolia and Kenya. In this article, two new strains of *Dethiobacter alkaliphilus*, initially described as sulfur/thiosulfate-reducing and iron-reducing bacteria, respectively, were both found to be capable of reducing iron and thiosulfate. Distinct sets of multiheme cytochromes that probably mediate these reactions were identified and their high variation was suggested to be an effective adaptive strategy for occupying geochemically diverse extreme environments. Analysis of the distribution of this genus in natural environments revealed strong connection to alkaline, sulfur- or iron-enriched ecotopes (soda lakes and serpentinite-associated sediments). Soda lakes are geologically ancient ecosystems that are still widespread on Earth and that are thought to harbor archaic microbial communities, but they might constitute secondary habitats for *D. alkaliphilus* when compared with Fe-rich serpentinites. These results increase our understanding of the metabolic diversity of extremophiles and point to evolutionary traits that could have occurred in prokaryotes during the early stages of the biosphere's evolution, namely when the sulfur biogeochemical cycle overtook the iron cycle as a predominant biogeochemical process.

The inspection of the extensive repositories in culture collections can also yield new taxa. The contribution by Montero-Calasanz et al. looks at isolates deposited at the Leibniz Institute DSMZ in the 1990s that originated from soils in the Atacama Desert, Chile, and landfill leachate from Vancouver, Canada. Analysis of these isolates, which belong to the *Geodermatophilaceae*, used new genomic data to determine genotype-phenotype correlations within this family. Based on their results, the authors describe and name four novel species in the genus *Blastococcus*, introduce the new genera *Trujillonella, Pleomorpha*, and *Goekera*, and reclassify *Blastococcus endophyticus* as *Trujillonella endophytica, Geodermatophilus daqingensis* as *Pleomorpha daqingensis*, and *Modestobacter deserti* as *Goekera deserti*. The authors also emphasize how genomics can effectively replace and/or complement some of the routine phenotypic analyses in microbial systematics.

Additionally, two articles from the Daroch group describe new genera of thermophilic cyanobacteria isolated from terrestrial hot springs and name them under the botanical code *Trichothermofontia sichuanensis* (Tang et al.) and *Thermocoleostomius sinensis* (Jiang et al.). Both articles also investigate carbon-concentrating mechanisms based on annotations of bicarbonate transporters, carbonic anhydrases, and carboxysome-associated genes. For *Thermocoleostomius sinensis*, these genes were also shown to be expressed under low-CO_2_ conditions.

## Community-based studies

The use of molecular methods to analyze microbial community structure and functional diversity in different environments continues to be a formidable tool. This volume includes new community-based studies in previously overlooked or understudied extreme and poly-extreme environments in Africa, Asia, Europe, and the Americas.

In one of the contributions, Gawas and Kerkar study sediments from three salt pans, adjoining different prominent estuaries in Goa, India. Marked differences in the unique genera found for each site led the authors to suggest that the different estuaries select for unique bacterial diversity.

In another publication, an amplicon sequencing study in the East African Rift Valley analyzes prokaryotic and eukaryotic diversity in three soda lakes, Lake Abijata, Lake Chitu, and Lake Shala, and compares the results with enrichment culture methods (Jeilu et al.). The culture-independent approach captured higher diversity than previously detected in these lakes, provided a more reliable estimate of microbial diversity than traditional culture methods, pointed to differences in microbial community composition among the three lakes, and identified the most commonly found prokaryotic taxa—*Pseudomonadota* (*Halomonas*), *Bacillota* (*Bacillus, Clostridia*), *Bacteroidota* (*Bacteroides*), *Euryarchaeota* (*Thermoplasmata, Thermococci, Methanomicrobia, Halobacter*), and *Nanoarchaeota* (*Woesearchaeia*)—and eukaryotic taxa—*Ascomycota* and *Basidiomycota*.

In a different poly-extreme setting, Zhang et al. assess the prokaryotic taxonomy and potential functional diversity of three hyper-arid soil samples from the Gobi Desert. Their study compares two different next-generation sequencing platforms and identifies 36 bacterial phyla, including *Pseudomonadota, Bacteroidota, Bacillota, Actinomycetota, Methanobacteriota, Acidobacteriota, Nitrososphaerota*, and *Planctomycetota*. The authors suggest that environmental factors such as total dissolved salts, available potassium, total nitrogen, and organic matter are positively correlated with the abundance of most groups, and that the community structure was mainly controlled by stochastic processes.

Another focus of this section is on metagenomes from geothermal systems. The study by Ugwuanyi et al. compares metagenomes from different substrates—water, mud, and fumarolic deposits—from Solfatara and Pisciarelli hydrothermal systems in Italy, and reveals a high abundance of pathways for carbon fixation and sulfur oxidation. A separate metagenomic study recovered and interpreted *Caldarchaeales* metagenome-assembled genomes from geothermal features in Hawai'i and Chile and proposes both chemoorganotrophic and chemolithotrophic lifestyles (Balbay et al.).

## New insights into high taxonomic ranks of extremophiles

The final highlights of this volume center on studies targeting high ranks in the taxonomy of extremophiles and the new insights that such studies can provide.

Looking into new frontiers within this field, Wu et al. delve into the archaeal class *Halobacteria* and provide an overview of astrobiology-relevant exposure experiments, identifying key knowledge gaps and research opportunities within this model group of microorganisms. The authors stress that further testing is still needed to fully cover the taxonomic diversity present in this archaeal group both at high and low ranks. This should be prioritized given the relevance of the group for the search for life on Mars and on the exooceans of the icy moons, for planetary protection, and for the expected future needs of the space biotechnology sector.

The final contribution looks at the *Halomonadaceae*, the most diverse family of halophilic bacteria, and a group in dire need of an in-depth taxogenomic analysis. The study by de la Haba et al. delivers on this daunting task by sequencing the genomes of 17 type strains and comparing them with all other publicly available genome sequences within this family. Based on comparative genomic, phylogenomic, and clade-specific signature gene analyses, the authors suggest that the genus *Halovibrio* is misplaced within the family, propose a division of the genus *Halomonas* into seven separate genera with reclassification of some of its current species to the genus *Modicisalibacter*, and identify various synonymous species names within the family.

## Final remarks

The study of extreme environments and their microbial inhabitants is the subject of intense interest and activity by researchers across different expertise and spread across the globe. The next few years of research, supported by the increased use and further developments in genomics and taxogenomics, promise to continue to deliver exciting new advances on this disciplinary field with implications on different aspects of our lives on Earth and beyond.

## Author contributions

AA: Conceptualization, Data curation, Funding acquisition, Project administration, Supervision, Validation, Writing – original draft, Writing – review & editing. RRH: Conceptualization, Data curation, Funding acquisition, Project administration, Supervision, Validation, Writing – original draft, Writing – review & editing. MJ: Data curation, Funding acquisition, Project administration, Supervision, Validation, Writing – original draft, Writing – review & editing. BPH: Conceptualization, Data curation, Project administration, Supervision, Validation, Writing – original draft, Writing – review & editing.
